# Recent advances in understanding lymphangiogenesis and metabolism

**DOI:** 10.12688/f1000research.14803.1

**Published:** 2018-07-20

**Authors:** Heon-Woo Lee, Pengchun Yu, Michael Simons

**Affiliations:** 1 Yale Cardiovascular Research Center, Section of Cardiovascular Medicine, Department of Internal Medicine, Yale University School of Medicine, New Haven, CT, USA

**Keywords:** lymphatic system, lymphangiogenesis, metabolism, homeostasis, blood and lymphatic vasculatures

## Abstract

The blood and lymphatic vasculatures are vital to the maintenance of homeostasis. The interaction between two vascular networks throughout the body is precisely controlled to enable oxygen and nutrient delivery, removal of carbon dioxide and metabolic waste, drainage of interstitial fluid, transport of immune cells, and other key activities. Recent years have seen an explosion of information dealing with the development and function of the lymphatic system. The growth of lymphatic vessels, termed lymphangiogenesis, is a high-energy requirement process that involves sprouting, proliferation, migration, and remodeling of lymphatic endothelial cells and capillaries. Although there has been substantial progress in identifying growth factors and their downstream signaling pathways that control lymphangiogenesis, the role of metabolic processes during lymphangiogenesis and their links to growth factor signaling are poorly understood. In this review, we will discuss recent work that has provided new insights into lymphatic metabolism and its role in lymphangiogenesis.

## Introduction

Adenosine 5'-triphosphate (ATP) is the main carrier of cellular energy and its generation is tightly coupled to metabolic demand, ensuring sufficient energy for cellular growth, repair, movement, and other biological processes ^1^. The three principal sources used by mammalian cells to generate ATP molecules are fats, proteins, and carbohydrates ^2^. These three nutrients are referred to as “mitochondrial nutrients” because they are essential commodities to yield ATP in mitochondria ^3^. This process of generation of ATP in the mitochondria is referred to as oxidative phosphorylation, since it involves phosphorylation of ADP (to produce ATP) as a result of electron transfer from NADH or FADH _2_ to O _2_ by a series of oxidative reactions ^4^. The end product is the generation of 32 ATP molecules from one molecule of glucose ^5^. In addition to oxidative phosphorylation, ATP also can be generated anaerobically by glycolysis. During glycolysis, one molecule of glucose is converted into two molecules of lactate with the production of two molecules of ATP ^6^. Traditionally, it has been assumed that normal cells rely predominantly on mitochondrial oxidative phosphorylation to yield most of the ATP needed for cellular work ^7^. As will be described below, the situation in the endothelium, both blood and lymphatic, is quite different.

The lymphatic vasculature serves a number of key functions, including regulation of interstitial fluid homeostasis and immune cell transport ^8^. The majority of lymphatic endothelial cells (LECs) are derived from the anterior cardinal vein endothelial cells (ECs) around embryonic day 9.5 ^9, 10^, although there are alternative sources for LECs in the skin ^11^, heart ^12^, and intestine ^13^. During lymphangiogenesis, the primary lymphatic plexus is reorganized into lymphatic capillaries, lymph nodes, and collecting lymphatic vessels ^14, 15^. Lymphatic capillaries serve as entry points for the interstitial fluid that then is transported, via collecting lymphatic vessels, back to the blood circulation via the thoracic duct that drains into the left subclavian vein. Similarly, immune cells enter the lymphatics via its open-ended capillaries and then are transported to regional lymph nodes ^16^. Not surprisingly, all processes involved in lymphatic vasculature specification, formation, and maintenance, as well as performance of normal everyday activities, require energy. Recent advances have led to a better understanding of lymphatic metabolism and their contribution to lymphangiogenesis, which is the subject of this review.

## Metabolic environment of lymphatic endothelial cells

The lymphatic endothelium exists in a unique metabolic environment. The lymphatic fluid is rich in nutrients, even though it is the product of drainage of fluid waste from peripheral tissues. Typically, LECs are exposed to high concentrations of glucose (4–6 mM) ^17, 18^, total protein (8–32 g/L) ^17^, and triglycerides (8–40 mg/dL) ^17, 18^. At the same time, oxygen concentration in the lymphatic fluid in the thoracic duct and capillary lymphatics is relatively low with partial pressure of oxygen (pO _2_) 15–42 mmHg in the lymph fluid compared with 80–100 mmHg in the arterial blood ^19– 22^. This environment may, to an extent, account for the metabolic peculiarities of the lymphatic endothelium.

## Glucose metabolism in lymphatic endothelial cells

**Figure 1.**
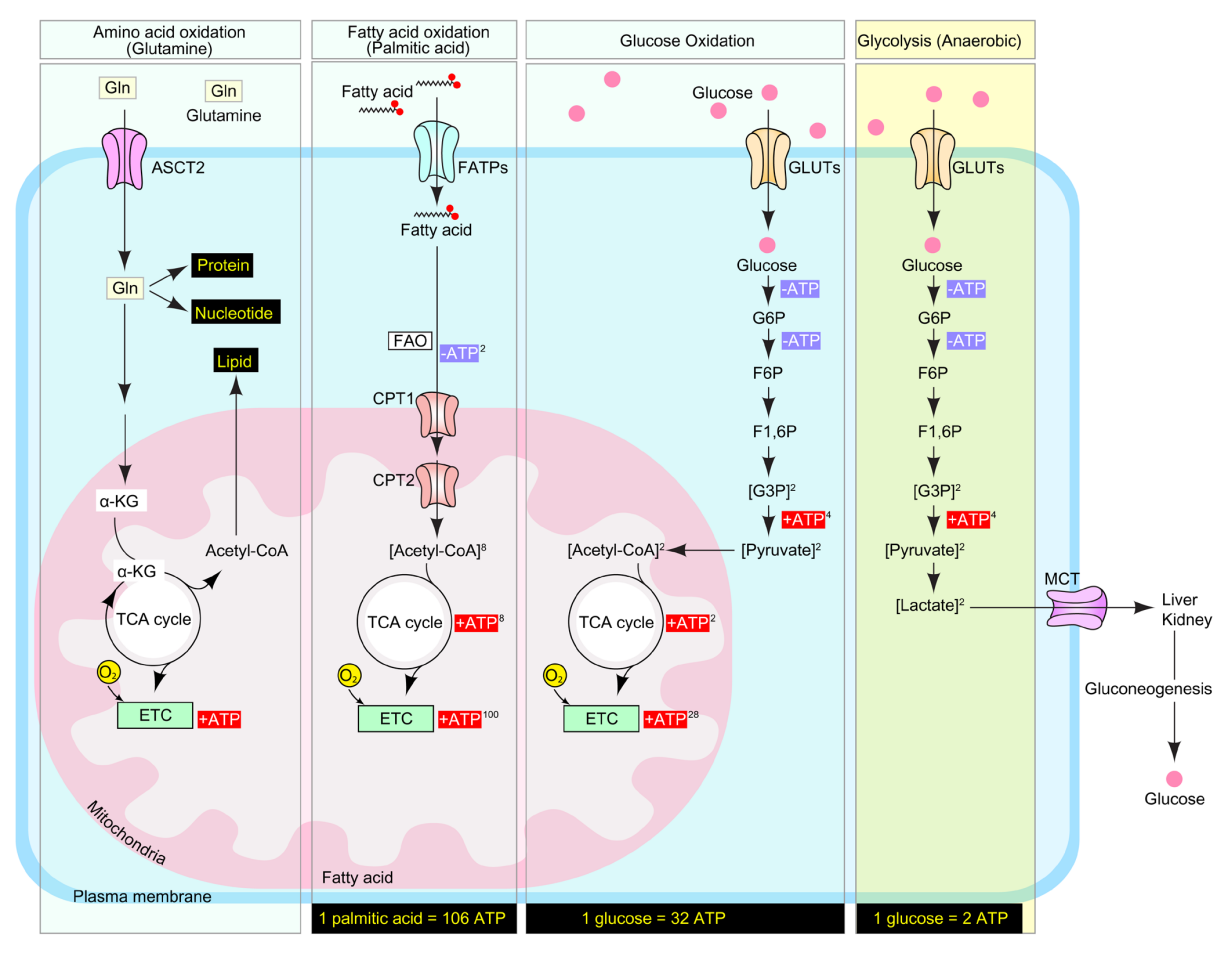
Adenosine 5'-triphosphate generation in mammalian cells. Amino acids (glutamine), fatty acids (palmitic acid), and carbohydrates (glucose) are the main sources of adenosine 5'-triphosphate (ATP) in the mammalian cell. Glutamine enters the mammalian cell through a glutamine transporter such as ASCT2. Glutamine serves as a precursor for the synthesis of lipids, nucleotides, proteins, and ATP. Fatty acids (palmitic acid) are transported into cytosol by FATPs (fatty acid transporters) and collapsed into acetyl-CoA through FAO (fatty acid oxidation). CPT1 (carnitine palmitoyltransferase I) on the mitochondrial outer membrane and CPT2 (carnitine palmitoyltransferase II) on the inner membrane cooperate for FAO. During glucose oxidation, glucose is transported across the cell membrane by GLUTs (glucose transporters) and catabolized into pyruvate. Pyruvate then enters the mitochondria and is converted into acetyl-CoA, which initiates the tricarboxylic acid (TCA) cycle to generate ATP. During anaerobic glycolysis, glucose is catabolized into pyruvate by using the same mechanism of glucose oxidation, but pyruvate turns into lactate and is finally exported into the bloodstream by monocarboxylate transporter (MCTs). During this metabolic process, complete oxidation of one molecule of nutrient generates 106 ATP (palmitic acid), 32 ATP (glucose; aerobic), and 2 ATP (glucose; anaerobic) molecules. ATP, adenosine 5'-triphosphate; ETC, electron transport chain.

Glucose is the principal energy source in the endothelium ^23^. Having entered the cell, it can undergo either oxidative phosphorylation, a catabolic process involving its conversion into carbon dioxide and water, or anaerobic glycolysis that leads to the production of lactate. When coupled with mitochondrial respiration, one molecule of glucose produces 32 ATP molecules upon complete oxidation ^24^ ( Figure 1). Alternatively, anaerobic glycolysis, which takes place in the cytosol and does not involve the consumption of oxygen, leads to the production of two molecules of ATP during glucose-to-lactate conversion ( Figure 1). Initially, it was thought that most normal, healthy cells predominantly employ glucose oxidation rather than glycolysis and that the latter is reserved for anaerobic conditions ^7, 25– 27^. However, many types of cancer cells as well as certain normal cell types, including astrocytes ^27, 28^, fibroblasts ^29, 30^, activated T cells ^31^, and pro-inflammatory macrophages ^31^, depend predominantly on glycolysis for their energy demands.

More recently, several studies documented that blood vasculature ECs also rely primarily on anaerobic glycolysis for their energy generation ^32– 34^. Studies of glycolytic flux using radiolabeled glucose show that 99% of glucose is converted to lactate, which is a final product of glycolysis, and only a very small fraction (~0.04%) of pyruvate enters the mitochondria for glucose oxidation in rat coronary ECs ^32^. LECs have also been reported to take up massive amounts of glucose compared with non-endothelial healthy cells and to use glycolysis as the primary source of ATP (~85%) with mitochondrial glucose oxidation almost completely inactive ^23^. One potential explanation for this preference is that anaerobic glycolysis allows the production of energy at sites of cell locomotion (that is, lamellipodia and filopodia formation), thereby avoiding the need for ATP transport from the mitochondria ^35^. Another is that interstitial tissue fluid which is absorbed into lymphatic vessels is the end product of somatic oxidative metabolism with low oxygen concentration ^19– 22^, and glycolysis maximizes the efficiency for ATP synthesis under their distinct nutritional environment.

## Fibroblast growth factor signaling and glucose metabolism

As its name indicates, fibroblast growth factors (FGFs) were first identified as regulators of fibroblast proliferation ^36, 37^. Over the past four decades since the first purification and identification of FGFs, it has become clear that FGF signaling is not limited to fibroblasts but occurs in most cell types. Furthermore, it is involved with numerous normal ^38^ and pathological conditions ranging from development ^39– 41^ and tissue homeostasis to coronary artery disease ^42^, scleroderma ^43^, hypercholesterolemia ^44^, pulmonary arterial hypertension ^45, 46^, and atherosclerosis ^47, 48^, among many others.

Recent studies demonstrated an important role played by the FGF signaling cascade in lymphatic development that, in many ways, is as critical and important as a better recognized contribution of vascular endothelial growth factor (VEGF) signaling ^49– 53^. FGF ligands initiate their signals through high-affinity tyrosine kinase receptors (FGFR1–4) and a number of co-receptors, including syndecan-4 and Klotho ^54^. There is a high level of redundancy between the 22 FGFs and four FGFRs, leading to frequent compensatory effects. One example of that is the paucity of vascular developmental abnormalities in *Fgfr1* or *Fgfr3* single-knockout ^55^ and *Fgfr1/Fgfr2* double-knockout ^56^ mice. These phenotypes come to the fore, however, in mice with disrupted expression of both *Fgfr1* and *Fgfr3* genes ( *Fgfr1* ^fl/fl^; *Fgfr3* ^−/−^ with Prox1[BAC]-CreER ^T2^) ^55^. These animals show impairment of LEC migration and lymphatic vessel branching, resulting in significant edema, appearance of blood-filled lymphatics, and reduced dermal lymphatic development ^55^. RNA-seq analysis of gene expression in LECs before and after FGF2 or small interfering RNA (siRNA)-mediated disruption of FGF signaling showed that FGFs regulate not only proliferation and migration but also metabolic processes in LECs, glucose metabolism in particular ^55^. Among the genes involved in glucose metabolism, *HK2* (hexokinase 2) was identified as the highest-ranking gene regulated by FGFs ^55^.

**Figure 2.**
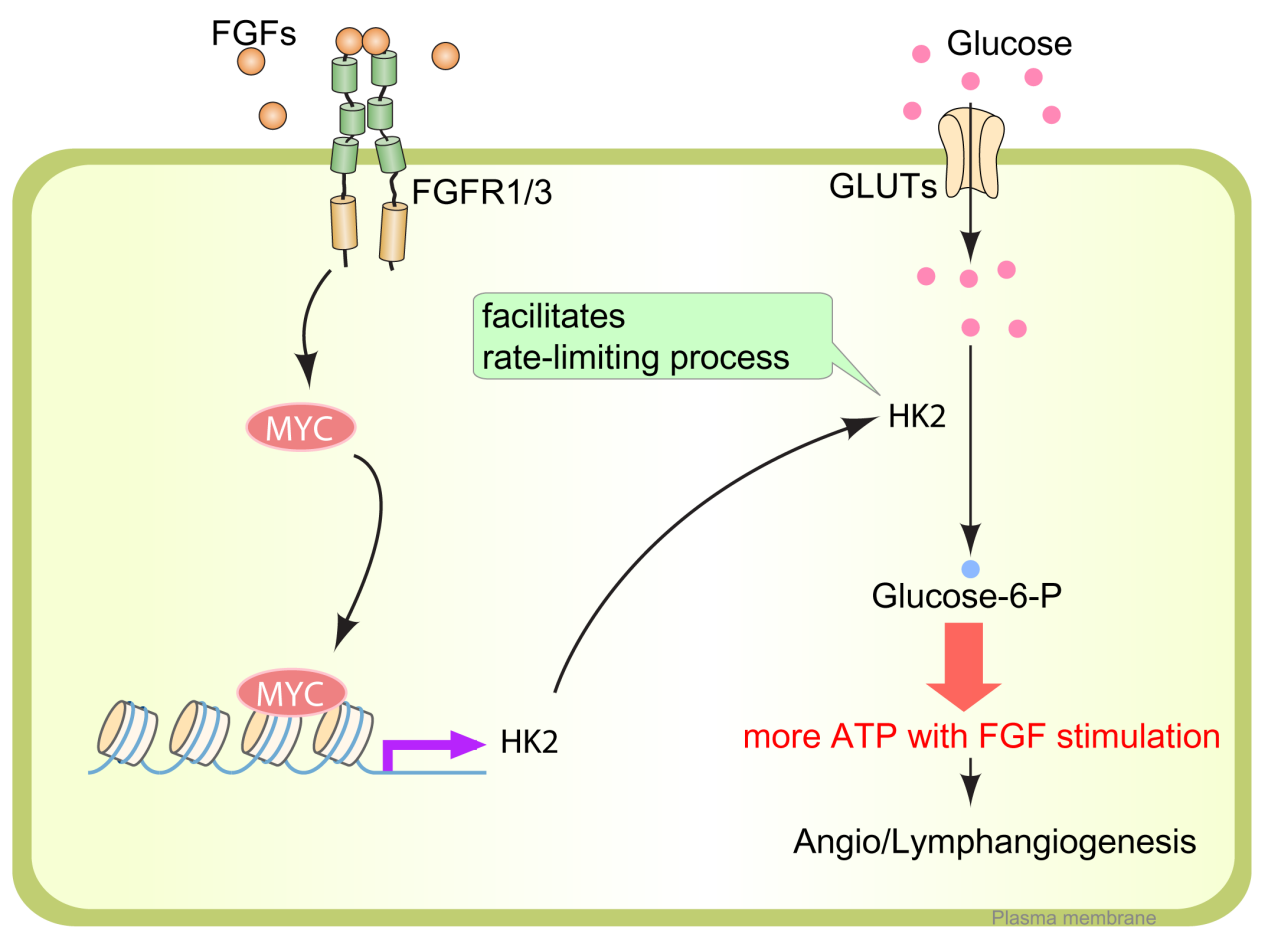
Fibroblast growth factor signaling and lymphatic endothelial metabolism. After fibroblast growth factor (FGF) ligand binding, FGFR1 and FGFR3 initiate a signaling cascade and induce MYC expression in LECs. MYC binds to the regulatory region of the *HK2* gene, a rate-limiting enzyme catalyzing the first step of glucose breakdown into glucose-6-phosphate. The FGFR1/3–MYC–HK2 axis is a crucial driver of glycolysis for LECs to generate adenosine 5'-triphosphate (ATP), which is needed for lymphangiogenesis. FGFR, fibroblast growth factor receptor; GLUT, glucose transporter; HK2, hexokinase 2; LEC, lymphatic endothelial cell.

Hexokinases (HKs) are rate-limiting enzymes catalyzing the first step of glucose breakdown, which is the ATP-dependent phosphorylation of glucose to glucose 6-phosphate (G6P) ( Figure 2). Among their four isoforms (HK1–HK4), HK2 is a predominant isoform in insulin-sensitive cells such as cardiomyocytes, skeletal muscle, and adipocytes; it is also upregulated in many types of tumors, and its expression is associated with enhanced glycolysis and tumor growth ^57^. HK2 deletion in either all or lymphatic-only ECs in mice using, respectively, Cdh5(PAC)-CreER ^T2^ or Prox1(BAC)-CreER ^T2^ showed reduced glycolysis and retarded blood or lymphatic vascular development (or both) in various organs, including skin, cornea, and retina ^55^, supporting the importance of HK2.

One of the factors that regulate HK2 expression is hypoxia. Under hypoxic conditions, hypoxia-inducible factor-1 alpha (HIF1α) expression is increased and, in turn, enhances the expression of the *HK2* gene through binding to the E-box sequence 5'-CACGTG-3' in the HK2 promoter in cooperation with MYC transcription factors ^58, 59^. In cell types dependent on oxidative phosphorylation, this mechanism can result in a shift to anaerobic glycolysis, thereby allowing the cells to survive in hypoxic conditions ^58, 59^. Interestingly, activation of FGF signaling in LECs also enhances their glycolytic flux through MYC transcription factor expression ^55^ ( Figure 2). Reversely, suppression of FGF signaling input, including FGFR1 siRNA treatment or *Fgfr1/Fgfr3* deletion in LECs, leads to reduced expression of MYC and HK2 and a suppression of glycolysis ^55^ ( Figure 2).

The importance of this “metabolic” aspect of FGF signaling is emphasized by the fact that an LEC-specific deletion of HK2 leads to a very similar phenotype with *Fgfr1/Fgfr2* double-knockout mice ^55^. That the metabolic process could be manipulated through external growth factor-initiated signaling in LECs is intriguing because it suggests that the metabolic pathway could be a therapeutic target for lymphangiogenesis-related diseases.

## Fatty acid oxidation in lymphatic endothelial cells

**Figure 3.**
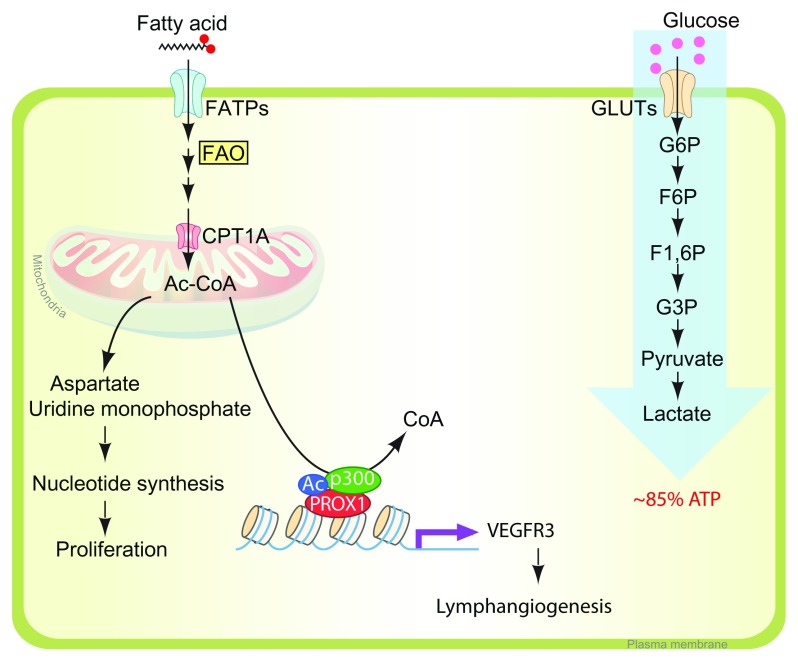
Metabolism on lymphatic endothelial cells. In lymphatic endothelial cells (LECs), fatty acid is used primarily for nucleotide biosynthesis. During the tricarboxylic acid (TCA) cycle in mitochondria, acetyl-CoA provides the carbon skeletons for aspartate and uridine monophosphate. These intermediates are used in nucleotide synthesis in proliferating LECs. Acetyl-CoA catabolized from fatty acid is also used for histone acetylation of VEGFR3 promoter region with PROX1/P300 protein complex. (Left) The major source of ATP in LECs is glucose. (Right) Up to 85% of ATP is generated using glycolysis (anaerobic). ATP, adenosine 5'-triphosphate; CPT1A, carnitine palmitoyltransferase 1A; F1,6P, fructose 1,6-diphosphate; F6P, fructose-6-phosphate; FAO, fatty acid oxidation; FATP, fatty acid transporter; G3P, glucose 3-phosphate; G6P, glucose 6-phosphate; GLUT, glucose transporter; VEGFR3, vascular endothelial growth factor receptor 3.

Fatty acids are an important nutrient source not only for energy generation but also for biomass synthesis necessary to support cell proliferation. Carbon and nitrogen derived from fatty acid oxidation (FAO) become the building blocks for nucleotide synthesis, which is necessary for DNA replication and transcription. Although in most cell types glucose and glutamine supply most of the carbon and nitrogen necessary to support cell growth and division ^25^, recent studies demonstrated that in ECs fatty acid carbon, and not glucose, is primarily used to support RNA and DNA nucleotide synthesis ^60^ ( Figure 3). Using radiolabeled palmitate, a 16-carbon saturated fatty acid, Schoors *et al*. showed that its carbon incorporates into aspartate (a nucleotide precursor) and uridine monophosphate (a precursor of pyrimidine nucleoside triphosphates and DNA) in human umbilical vein ECs ^60^ ( Figure 3). CPT1A (carnitine palmitoyltransferase 1A), an enzyme localized to the mitochondrial outer membrane, controls the translocation of fatty acids across the mitochondrial membrane and acts as the rate-limiting regulator of FAO. An endothelial-specific knockout of *Cpt1a* in mice ( *Cpt1a* ^fl/fl^; Cdh5[PAC]-CreER ^T2^) displayed reduced proliferation in stalk ECs, while leading tip cells are intact ^60^, suggesting that FAO stimulates vessel sprouting through EC proliferation. Analogous to blood ECs (BECs), LECs also use fatty acid as the principal source of carbon for nucleotide biosynthesis, but the FAO flux in LECs is even higher than in BECs while glycolytic flux is lower, implying that LECs use a metabolic pathway distinct from that of BECs ^61^.

## Molecular signaling and fatty acid metabolism

A critical step in lymphangiogenesis is the specification of lymphatic endothelial fate. During embryonic development, around embryonic day 9.5, a small subset of anterior cardinal vein ECs begin expressing a lymphatic marker, Prox-1, which is driven by ERK-dependent activation of transcription factor Sox18 expression ^15, 62^. This leads to venous-to-endothelial cell fate transition and activation of expression of LEC-specific markers, including LYVE-1, PROX1, FGFR3, and VEGFR3 ^62, 63^. PROX1-expressing LECs migrate into the surrounding tissue to form primitive lymph sacs and lymphatic plexus ^15^. PROX1 regulates the lymphangiogenic receptor tyrosine kinase VEGFR3 expression, which is responsible for LEC migration and proliferation. The absence of PROX1 expression results in the complete loss of the lymphatic system and decreased VEGFR3 expression in developmental lymphangiogenesis ^15^.

In addition to the PROX1-mediated molecular signaling pathway, a recent study showed that PROX1 is involved with FAO metabolism for lymphangiogenesis. In PROX1-deficient cultured LECs, CPT1A expression and FAO flux are also inhibited, and this led to inhibition of proliferation. Consistent with these findings, LEC-specific *Cpt1* deletion in developmental mouse embryo ( *Cpt1* ^fl/fl^ with Prox1[BAC]-CreER ^T2^) also showed defective edema caused by impaired LEC proliferation, migration, and sprouting ^61^. Interestingly, FAO inhibition through CPT1KD decreased VEGFR3 expression, which suggests that FAO is essential not only for fatty acid metabolism but also for maintaining LEC identity through transcriptional gene regulation. Mechanistically, overexpression of PROX1 upregulates CPT1A transcription, which increases the generation of acetyl-CoA through fatty acid β-oxidation ^61^ ( Figure 3). Acetyl-CoA is used for two different purposes on LECs. First, it provides a carbon source for nucleotide synthesis, which eventually leads to LEC proliferation ^61^. Second, LEC uses acetyl-CoA for histone acetylation of VEGFR3 promoter region with PROX1 and p300 (histone acetyltransferase p300) protein complex ^61^ ( Figure 3). Prox1 has also been shown to function as a metabolic transcriptional regulator in non-ECs. In liver cells, Prox1 interacts with ERRα/PGC-1α complex, which plays a central role in the maintenance of energy homeostasis and inhibits its activity ^64^. In colon cancer cells, Prox1 controls metabolic adaptation of these cells, promotes resistance to nutrient deprivation, and fuels metastatic outgrowth of tumors ^65^. With a chip-on-chip assay with Prox1, Prox1 was shown to target genes highly enriched for processes linked to bile acid biosynthesis and histidine metabolism ^64^. This suggests that metabolic regulation might be one of the major activities of Prox1.

VEGFR3 is a critical and indispensable regulator of lymphatic development and function. Its almost exclusive expression in LECs makes them respond to vascular endothelial growth factor-C (VEGF-C) for LEC proliferation, migration, and sprouting ^66^. VEGFR3 expression is known to vary with developmental stage and pathological conditions. It is expressed in BECs during early embryogenesis, but later, VEGFR-3 expression becomes restricted to the LECs. Vegfr3 knockout mice die because of angiogenic defect around embryonic day 10.5, before the establishment of the lymphatic system ^67^. Heterozygous functional null mutations in the Vegfr3 gene which inactivate the tyrosine kinase do not cause any defect in embryonic angiogenesis, but they have been linked to lymphedema ^68, 69^. Dynamic changes in the endogenous expression level of VEGFR3 indicate that VEGFR3 transcription is precisely regulated. VEGFR expression can be altered by numerous transcription factors, including SP1, SP3, ETSs, p300, PROX1, and E2F ^70– 72^, and by epigenetic modification of the regulatory sequences, including histone acetylation and CpG methylation ^72, 73^. Among the genes mentioned, ETSs, Prox1, and p300 are involved in histone acetylation of the *Vegfr3* gene, which likely plays a role in its transcriptional regulation ^70– 72^. This combination of events leads to fatty acid-derived acetyl-CoA, which plays a role in histone acetylation on the VEGFR3 promoter and regulation of VEGFR3 expression. Intriguingly, supplementation of acetate (a precursor of acetyl-CoA) is able to rescue lymphangiogenesis induced by CPT1A inhibition, implying the potential of metabolites for use in the field of regenerative medicine ^61^. Although FAO plays crucial roles during the lymphangiogenesis process, it is not yet clear why FAO serves as the main carbon source for nucleotide synthesis in LECs even though the lymph fluid provides abundant nutrients, including glucose and amino acids ^17, 18^, which are employed for biomass synthesis in other tissues and whether FAO-derived acetyl-CoA also serves for acetylation modification of other lymphatic genes or proteins.

## Perspective

In 1996, Joukov *et al*. purified and identified a cDNA that has VEGFR3 binding affinity and named it VEGF-C ^74^. Over the past two decades since the identification of VEGF-C, several additional molecular pathways, including transcription factors, receptors, and mediators, have been shown to regulate lymphatic differentiation and lymphangiogenesis. In recent years, different lines of work have led to the conclusion not only that the molecular signals are important to control lymphangiogenesis but also that metabolism could be an alternative target. More detailed understanding of the metabolic pathway in LECs will open new avenues for developing new therapeutic targets for lymphangiogenesis-related diseases.
